# 肺结节二维与三维体积测量的观察者内重复性比较

**DOI:** 10.3779/j.issn.1009-3419.2014.04.08

**Published:** 2014-04-20

**Authors:** 小皖 郭, 颖 王, 东 李, 翀 张, 阳 曹, 大同 苏, 铁链 于

**Affiliations:** 300052 天津，天津医科大学总医院放射科 Department of Radiology, Tianjin Medical University General Hospital, Tianjin 300052, China

**Keywords:** 肺结节, 计算机断层扫描, 体积测量, 重复性, Pulmonary nodule, Computed tomography, Volumetric measurement, Repeatability

## Abstract

**背景与目的:**

未定性肺结节的随访需要精确测量结节体积确定其生长特性。结节体积的三维测量可通过软件实现并应用于临床，其在临床实践中的价值尚需进一步验证。本研究回顾性分析肺结节患者胸部CT平扫影像资料，比较三维体积测量与传统二维肺结节测量的观察者内的重复性。

**方法:**

对2011年1月-2012年12月间在天津医科大学总医院行未定性肺结节CT随访研究的79例患者共86个结节的CT影像资料进行分析。由一名放射科医师对肺结节间隔1周行重复二维及三维体积测量。二维（two dimension, 2D）测量结节轴位最大横截面上的最大径（X）、相应垂直径（Y）及结节的头尾径（Z），分别根据球体及椭球体模型体积计算公式计算结节体积。三维（three dimension, 3D）测量通过计算机肺结节半自动体积测量软件进行，对结节自动体积分割效果不佳者行人工调整。应用*Logistic*回归分析评估结节的形态及位置对肺结节三维体积分割结果的影响。应用方差分析、相关分析评估3种体积测量方法的差异、*Bland-Altman*法评估3种方法的重复性。重复性定义为两次测量之间的相对差值（relative difference, RD）。

**结果:**

86例结节两次三维软件体积测量中，软件分割效果满意结节占81.4%。*Logistic*回归分析提示边缘不规则结节及与血管相连结节软件分割不满意的比率明显增高，似然比（odds ratios, OR）分别为4.0、4.5。方差分析显示经二维测量与三维软件体积测量所得体积具有明显差异（*F*=6.5, *P*=0.012），同一方法两次重复测量结节体积间无统计学差异（*F*=1.813, *P*=0.182）。软件测量体积与椭球体模型体积相关性较球体模型高（相关系数分别为0.974、0.882）。3D软件体积测量重复性最佳，RD 95%一致性区间为-14%-11.6%，其次为2D椭球体模型体积(-37.7%-39.9%)，最后为2D球体模型（-44.63%-46.4%）。

**结论:**

肺结节软件三维体积测量较二维测量具有更高的重复性。对软件体积分割不满意结节，包括不规则形态及与血管相连结节，我们建议测量结节的三维径线并应用椭球体模型计算体积。

## Abstract (en)

**Background and objective:**

Software oriented three-dimensional (3D) volumetric measurement of pulmonary nodules has been feasible in the follow-up of indeterminate pulmonary nodules, however, its value need a further validation. The purpose of this study is to retrospectively analyze the chest CT data of patients with pulmonary nodules to compare the intra-observer variability of 3D and two-dimensional (2D) volumetric measurement.

**Methods:**

Eighty-six pulmonary nodules in chest CT scans of 79 subjects were retrospectively analyzed. One radiologist measured the nodules twice with a 7 days interval using 2D and 3D methods respectively. The maximal diameter (X), the perpendicular diameter (Y) on maximal cross sectional area of the nodule and the caudo-cranial diameter (Z) were measured and the volume was calculated by two models: spherical and elliptical model. The 3D measurements were acquired with semi-automated software with manual adjustment on unsatisfied nodule segmentation. *Logistic* regression analysis was performed to evaluate the effect of nodule location and morphology on 3D nodule segmentation. *ANOVA* and correlation test were used to evaluate the difference among three methods. *Bland-Altman* method was applied to quantify the intra-observer variability.

**Results:**

Software achieved satisfied segmentation for 86.4% nodules. The irregular and juxtavacular nodules have significantly high odds rations (OR) of unsatisfied segmentation as 4.0, 4.5, respectively. The volume measured by three method was significantly different (*F*=6.5, *P*=0.012), while the repeated measurements did not led to significant difference (*F*=1.813, *P*=0.182). The Spearman correlation efficient between 3D volume and 2D volume with sphere and ellipsoid model was 0.97, 0.88. The 95% limits of agreement of RD between two repeated measurements were -14%-11.6%, -37.7%-39.9% and -39.8%-45.8% for 3D, 2D with elliptical model and spherical model, respectively.

**Conclusion:**

The 3D volume measurement of pulmonary nodules is more repeatable than 2D volume measurement. Unsatisfied segmentation can occurred on a small number of nodules, especially for irregular and juxtavascular nodules. For these nodules, the measurement of 3D diameters is recommended.

在世界范围内，肺癌目前所导致的癌症死亡在所有肿瘤中居于首位^[1]^。早期发现、治疗可以明显提高肺癌的5年生存率^[2]^。随着计算机断层扫描技术提高及其高分辨率的优势，大量的肺小结节在检查中被发现^[3]^。恶性结节常表现为持续快速增长的特征^[4]^，对肺内定性困难的结节进行随访确定其生长性是目前临床常采用的策略。因此，精确测量结节体积对于肺癌的早诊早治至关重要。

目前，计算机体积测量软件已经逐步应用于肺癌筛查及临床实践中^[5-8]^，相对于传统二维体积测量而言, 具有更好的可重复性。现有的众多研究多针对肺结节计算机三维体积测量技术的准确性及可重复性^[9-16]^，但很少直接对肺结节的二维及三维测量进行直接比较。因此，本研究目的是回顾性分析肺结节患者胸部CT平扫影像资料，比较肺结节三维体积测量与传统二维测量方法间观察者内的重复性，评估结节形态及位置对三维体积测量的影响。

## 1 材料和方法

### 1.1 临床资料

回顾性分析2011年1月-2012年12月在天津医科大学总医院放射科行64排螺旋CT检查发现肺结节并在我院进行随访的患者。入选条件：肺内无钙化结节，结节大小在3 mm-30 mm之间。共有79例符合条件的患者（男性50例，女性29例），年龄范围32岁-83岁，平均年龄61±10.7岁，符合条件的结节共86个，结节平均直径7.3 mm±3.3 mm，直径范围3 mm-23 mm。结节的特性包括其形态及位置，形态分为四种：边缘光滑、分叶、毛刺及不规则，位置分为3种：结节完全在肺实质内、与血管相贴、胸膜相贴（表 1）。

**1:** 86例肺结节形态、位置分布及其对软件体积分割效果的影响：*Logistic*回归分析 The effect of morphology and location on software segmentation in 86 pulmonary nodules: the result of *Logistic* regression analysis

Nodule characteristics	*n*	Successful segmentation (%)	Odds ratios (95%CI)	*P*
Location				
Purely intraparenchymal	38	90	1	
Pleural-attached	29	76	2.6 (1.0, 7.0)	0.06
Juxtavascular	19	63	4.5 (1.6, 12.5)	0.004
Morphology				
Smooth	38	88	1	
Lobulated	20	83	1.3 (0.4, 4.1)	0.64
Spiculated	4	75	2.3 (0.4, 13.7)	0.37
Irregular	24	63	4.0 (1.5, 10)	0.004

### 1.2 CT图像采集

所有的检查均在64排螺旋CT机（GE Light Speed）进行，扫描范围自胸廓入口至肺底部，患者一次吸气后屏气完成全肺扫描，螺旋扫描方式，120 kV、300 mA，螺距1.375:1，机架旋转一周时间0.4秒，显示野（field of view, FOV）360 mm，图像矩阵512×512，扫面层厚为5 mm，默认重建算法为标准算法，然后重建1.25 mm层厚轴位图像。

### 1.3 结节体积定量

二维及三维测量均在GE AW4.6工作站进行，由一位放射科医生间隔一周行2次测量。二维体积测量的方法：在1.25 mm层厚标准算法的横断面图像上选取结节最大层面，应用电子卡尺测量其最大径（X），相应垂直径（Y），并根据结节层厚的数目乘以1.25确定结节的头尾径（Z）。我们采用两种体积计算模型计算^[14, 15]^，一种为球体模型，体积计算公式为V=1/6^*^π^*^X^3^，另一种为椭球体模型，体积计算公式为V=1/6^*^π^*^X ^*^Y^*^Z。三维体积测量应用ALA（advanced lung analysis）软件进行。具体操作步骤：选择要进行容积定量的一组数据，进入容积分析界面后，在打开的横断面图像上由观察者用鼠标单击要分析的结节，软件自动实现对结节的分割并显示出结节的三维及轴位的分割图像并显示容积。操作者可在轴位图像观察软件的分割情况，对分割不满意的结节可以通过调整鼠标的选取点、设定结节周围提取范围（3 mm-30 mm）、根据结节形态及位置不同选择应用相应算法对结节的分割进行调整以达到最佳效果，如果经调整后结节分割的区域刚好完整的包绕肺结节，则结果为分割满意，否则确定为分割不满意（图 1）。

**1:**
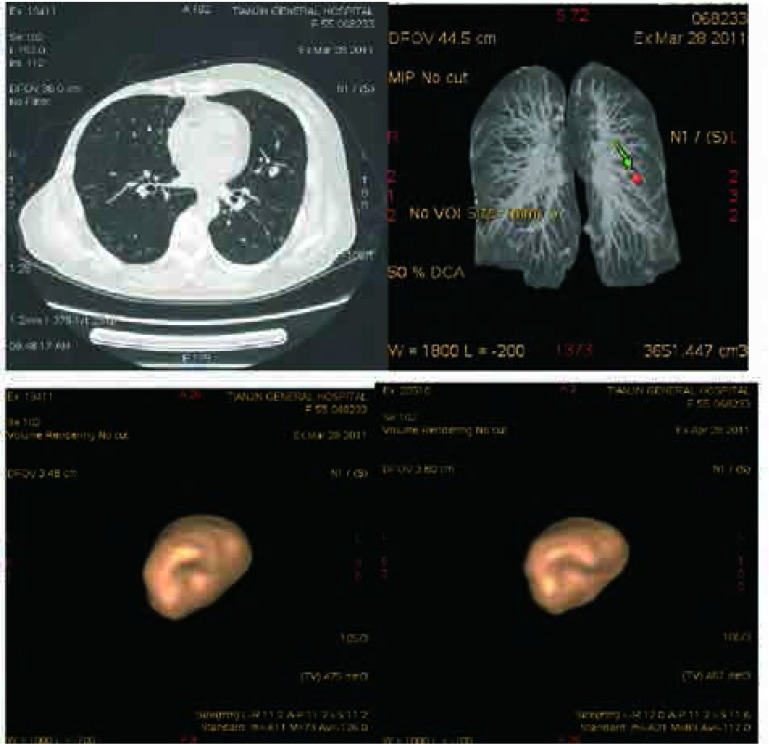
女性，55岁，查体发现左上叶结节（白色箭头），第一次容积测量结果显示结节容积为475 mm^3^，三维方向最大径线分别为（X、Y、Z）10.3 mm、7.3 mm、11.25 mm；第二次容积测量结果结节容积为467 mm^3^，三维方向最大径线分别为（X、Y、Z）10.0 mm、7.8 mm、11.25 mm Female, 55 years old, a nodule (white arrow) was incidentally detected in left upper lobe during a routine physical examination. The software-generated volume of the nodule was 475 mm^3^, and the largest diameter in X, Y, Z is 10.3 mm, 7.3 mm and 11.25 mm at first time; The software-generated volume of the nodule was 465 mm^3^, and the largest diameter in X, Y, Z is 10 mm, 7.8 mm and 11.25 mm at second time

### 1.4 统计分析

以结节三维软件分割效果作为应变量，结节形态及位置作为自变量，应用*Logistic*回归分析评估结节的形态及位置对肺结节三维体积分割结果的影响。应用方差分析评估三种体积测量方法之间的差异。应用*Spearman*相关分析确定三维体积测量和二维体积测量两种模式之间的相关性。计算二次重复测量的相对差值（relative difference，RD = V1/V2-1，V1为首次测量肺结节体积值，V2为间隔一周测量肺结节体积值），应用*Bland-Altman*法计算RD的95%一致性区间确定测量重复性。所有统计分析都在统计学软件SPSS 17.0中完成，*P* < 0.05定义为差异具有统计学意义。

## 2 结果

### 2.1 86例结节两次三维软件体积测量结果

81.4%（140/172）结节软件分割效果满意，18.6%（32/172）分割效果不满意。*Logistic*回归分析提示结节边缘不规则及与胸膜或血管相连软件分割不满意的比率明显增高，OR（odds ratios）分别为1.4、1.1、1.6，而结节其它特性对结节分割不具有明显影响（表 1）。

### 2.2 三维及二维体积测量的差异及相关性

二维球体模型体积测量、椭球体模型体积测量及三维体积测量方法结节体积间具有明显差异（*F*=6.5, *P*=0.012），同一方法两次重复测量结节体积间无明显差异（*F*=1.813, *P*=0.182）。三维测量体积与椭球体模型体积相关系数为0.974（*P* < 0.001），与球体模型体积相关系数为0.892（*P* < 0.001）（图 2）。

**2:**
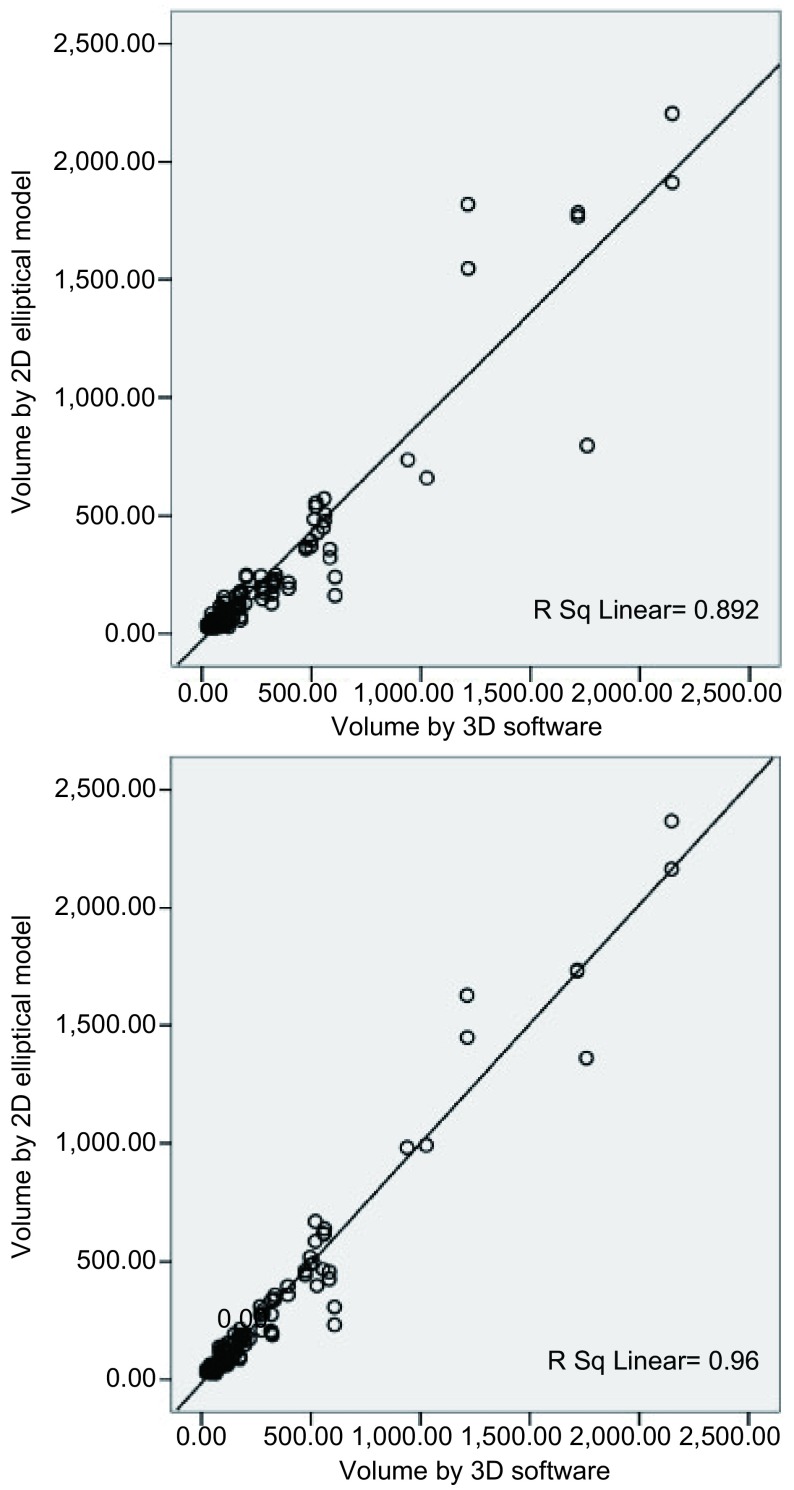
三维软件测量体积与二维球体模型、椭球体模型体积相关分析 The correlation between 3D software-oriented volume and 2D volume calculated by spherical and elliptical model

### 2.3 三维及二维体积测量的重复性

通过观察*Bland-Altman*散点图及三种体积估算方法二次测量RD的95%一致性区间发现，3D测量法的可重复性最好，其次为2D椭球体模型测量方法，2D球体模型测量法最差。分割满意结节3D体积测量RD均值-1.2%，95%一致性区间为-14%-11.6%，而2D椭球体模型体积RD均值为1.1%，95%一致性区间为-37.7%-39.9%，2D球体模型体积RD均值为1.5%，95%一致性区间为-39.8%-42.8%（图 3）。

**3:**
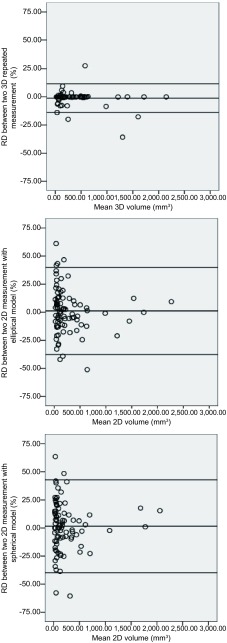
*Bland-Altman*分布图：两次体积测量之间的分布图：实线代表均值，虚线代表 95%一致性区间的上限及下限 The *Bland-Altman* plots of two repeated measurement with three methods. *Bland-Altman*: The dash line represents the mean Relative difference (RD), the dot line represents the upper and lower value of 95% limits of agreement

## 3 讨论

肺结节体积测量的准确性及可重复性对判断结节的良恶性有着决定性的作用，本研究通过回顾性分析的方法，比较同一观察者三种体积估算方法可重复性高低。选择最佳的体积估算方法，提高肺结节体积测量的可重复性，对肺结节的随访观察有着重要的临床意义。

体积测量一致性区间在结节的随访过程中对确定结节是否生长及生长率具有重要意义，我们研究发现，与二维容积测量相比三维体积测量方法具有很高的可重复性，其一致性区间几乎是二维体积测量方法的二分之一。一致性区间的上限在临床中可设定为结节是否生长的界限，假设应用三维体积测量方法，结节体积变化率为15%，超过95%一致性区间的上限，这时我们可以认为结节较前增长，而应用二维体积测量方法，15%仍位于可信区间之内，无法确定是由于测量的误差引起还是结节真正的生长引起。其次，容积倍增时间常用来量化肺结节在一定时间内的生长率，目前已经应用于肺癌筛查试验中，容积倍增时间小于400天已作为肺结节阳性生长的标准^[16]^。考虑到二维测量重复性较差，其在容积倍增时间的计算过程中受限：假设结节在3个月内二维测量方法观测到30%的体积增长，则容积倍增时间为237天，完全符合快速生长结节的定义，但根据我们的研究，此30%的增长完全可由于观察者内的差异引起。因此，三维体积测量是未来结节生长率量化的重要工具。

三维容积软件目前尚不完美，我们研究发现18%的肺结节利用软件分割并不令人满意，因此探寻潜在的负面影响因素并寻找解决方法就显得尤为重要。结节形态不规则及结节邻近支气管血管束时，结节分割较为困难，通过手动调节感兴趣区大小和（或）结节形状算法后仍有部分结节得不到满意分割。相反对于椭球体模型体积测量方法，结节的三条径线都是在观察者目测认为最佳的位置获得，通过椭球体体积计算公式得到的体积均可视为满意结节，但椭球体模型测量方法仍具有局限性，首先是目测径线的选取存在一定的随机性，其次结节本身形状与标准椭球体势必存在差异，这样就增加了结节测量结果的变异。相对于二维球体模型，椭球体模型体积测量方法需度量三个维度的径线，尤其对不规则结节来说，其更能反映结节的真实体积。

我们的结果和目前的研究结果相近。Wormanns等^[12]^利用计算机自动体积分割技术对肺内转移结节的体积进行定量时发现，短期内对肺结节进行体积定量，体积的变化为±20%，体积定量观察者内变异性是-3.9%-5.7%，变异性均较小。这与我们的研究结果一致，但未进一步分析肺结节位置的影响，值得一提的是他们研究的肺结节多为转移性结节，多为边缘光滑的实性结节。Iwano等^[17]^通过对60个确诊为周围型肺癌的结节进行肺结节计算机体积分割定量的重复性及潜在影响因素的研究中发现，结节体积、密度、边缘有无毛刺、是否邻近支气管血管束或邻胸膜之间的变异系数相差不大，但结节边缘模糊不清与结节边界清晰肺结节间的变异系数相差甚远（*P* < 0.01）。在最近的研究报道中，Kim等^[18]^在比较两种计算机分割软件对磨玻璃密度结节分割的可重复性及潜在危险因素中，发现肺结节邻近支气管血管束这一位置特点是分割软件均有的负面影响因素，Wang等^[19]^曾利用半自动体积分割技术分析肺结节形态特点变化对体积测量的影响，发现结节形态不规则及结节邻近支气管血管束对体积变化影响最大，他们的OR值分别为15.7、3.5，与我们分析得到的结果近似。

我们的研究也存在局限性，需要进一步克服。首先我们分析的是结节体积测量观察者内的变异性，而不同扫描方式之间测量的差异对容积也会有影响，例如患者每次的呼吸状态及位置、扫描位置不同所致部分容积效应等等，但由于对患者进行短期重复扫描具有放射损害，不符合伦理，利用肺体模结节的扫描将会帮助评估扫描之间的体积差异。其次，本研究结节样本量较小，还需进一步扩大以便得出最接近真实的结果。

总之，计算机三维体积估算方法在肺结节体积的测量中具有较高的可重复性，可以常规应用于肺结节生长性的评估，但体积分割的变异受结节位置，形态的影响，当三维体积分割无法得到满意的结果时可以通过二维测量结节三维径线，然后通过估计结节的形状，选择最接近的体积估算模型代替。
